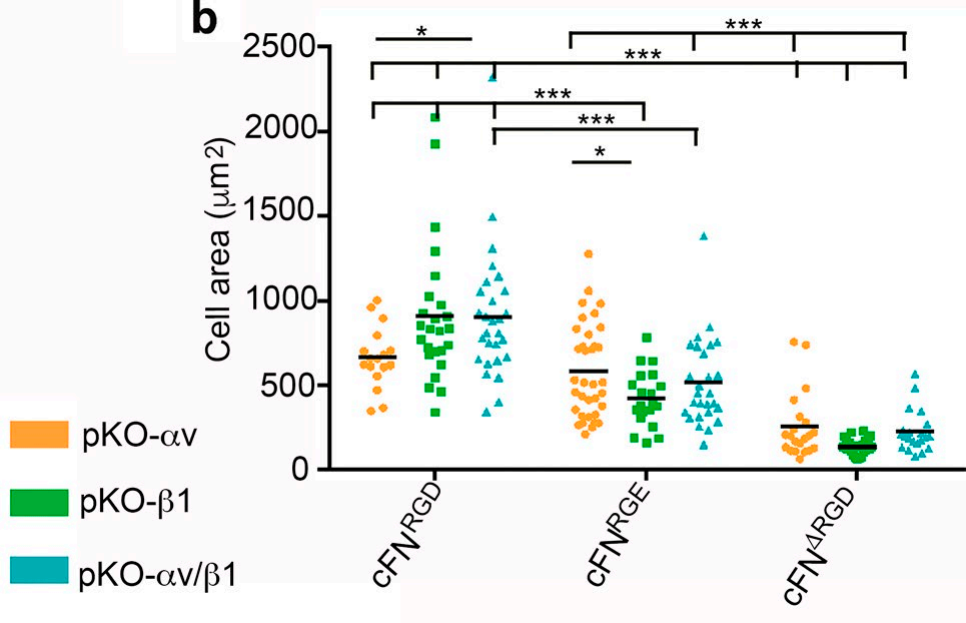# Correction: αv-Class integrin binding to fibronectin is solely mediated by RGD and unaffected by an RGE mutation

**DOI:** 10.1083/jcb.20200419812072020c

**Published:** 2020-12-11

**Authors:** María Benito-Jardón, Nico Strohmeyer, Sheila Otega-Sanchís, Mitasha Bharadwaj, Markus Moser, Daniel J. Müller, Reinhard Fässler, Mercedes Costell

After publication, an error was noticed in the "Cell spreading is altered by RGD mutations" section of the Results. The error occurred in the first paragraph, and the corrected text appears below.

"To test whether full-length cFN^RGE^ and cFN^ΔRGD^ allows α5β1 and αv-class integrin–mediated adhesion maturation and spreading, we plated pKO-αv, pKO-β1, and pKO-αv/β1 fibroblasts in serum replacement medium (SRM) on purified cFN variants and determined cell spreading as well as morphology and distribution of FAs (Fig. 2). At 60 min after seeding, the great majority of pKO-αv (95.8 ± 4.2%), pKO-β1 (93.1 ± 4.6%), and pKO-αv/β1 (98.0 ± 2.0%) fibroblasts spread on cFN^RGD^; a slightly lower percentage of pKO-αv (85.0 ± 5.6%) and pKO-αv/β1 fibroblasts (79.6 ± 7.9%) spread on cFN^RGE^; and a neglectable percentage of pKO-β1 fibroblasts spread on cFN^RGE^. On cFN^ΔRGD^, the numbers of spread pKO-αv and pKO-αv/β1 fibroblasts dropped to 22.2 ± 15% and 10.4 ± 7.0%, respectively, and of pKO-β1 fibroblasts to <1% (Fig. 2 a). Moreover, pKO-αv fibroblasts covered less spreading area than pKO-β1 or pKO-αv/β1 fibroblasts on cFN^RGD^ (744 ± 92 µm^2^ versus 897 ± 72 µm^2^, P = 0.022; and 906 ± 72 µm^2^, P = 0.018), similar to pKO-αv (583 ± 42 µm^2^) and pKO-αv/β1 fibroblasts (514 ± 49 µm^2^) on cFN^RGE^. The few adherent pKO-β1 fibroblasts covered a reduced area of 419 ± 36 µm^2^ (P < 0.05 compared to pKO-αv cells) on cFN^RGE^. In Fig. 2 b, statistical calculations change the significance of pKO-β1 compared to pKO-αv fibroblasts on cFN^RGE^ cells from P < 0.001 to P < 0.05. On cFN^ΔRGD^, the adherent pKO-αv, pKO-β1, and pKO-αv/β1 fibroblasts covered significantly smaller spreading areas (253 ± 42 µm^2^, 136 ± 8 µm^2^, and 227 ± 27 µm^2^, respectively) when compared with the same cell lines seeded on cFN^RGD^ (Fig. 2 b)."

The P values in Fig. 2 b have been adjusted accordingly, and the corrected figure panel is shown here. This change does not alter the conclusions of the manuscript. This error appears only in print and in PDF versions downloaded on or before December 11, 2020.

**Figure:**